# Effect of dietary supplementation of cinnamon oil and sodium butyrate on carcass characteristics and meat quality of broiler chicken

**DOI:** 10.14202/vetworld.2018.959-964

**Published:** 2018-07-18

**Authors:** Govindarajan Gomathi, Subramaniam Senthilkumar, Amirthalingam Natarajan, Ramasamy Amutha, Manika Ragavan Purushothaman

**Affiliations:** 1 Department of Animal Nutrition, Veterinary College and Research Institute, Tamil Nadu Veterinary and Animal Sciences University, Chennai, India; 2 Animal Feed Analysis and Quality Assurance Laboratory, Veterinary College and Research Institute, Tamil Nadu Veterinary and Animal Sciences University, Chennai, India; 3 Department of Poultry Science, Veterinary College and Research Institute, Tamil Nadu Veterinary and Animal Sciences University, Chennai, India

**Keywords:** antibiotic, broilers, carcass characteristics, cinnamon oil, coated sodium butyrate, meat quality

## Abstract

**Aim:**

An *in vivo* experiment was conducted to investigate the effect of supplementation of cinnamon oil (CO) and sodium butyrate on carcass characteristics and meat quality of broiler chicken compared with the antibiotic supplementation.

**Materials and Methods:**

A biological experiment was carried out with 216-day-old Vencobb-400 broiler chicks randomly distributed to six experimental treatments with six replicates, each replicate containing six chicks with equal numbers of male and female chicks. The experimental diets were prepared with isocaloric and isonitrogenous basis. The experimental groups, namely control (T_1_), control with antibiotic (T_2_), control with CO at 250 mg/kg and coated sodium butyrate (CSB) either at 0.09 (T_3_) or 0.18% (T_4_), and control with CO at 500 mg/kg and CSB either at 0.09 (T_5_) or 0.18% (T_6_). The trial was carried out in deep litter pen for 35 days. The carcass characteristics such as ready to cooked yield, eviscerated weight, heart, liver, gizzard, giblet, and abdominal fat percent in slaughtered birds and meat quality properties such as pH, water-holding capacity (WHC), tyrosine, shear force, cooking loss, thiobarbituric acid, sensory characteristics, and muscle cholesterol in breast muscle samples were evaluated.

**Results:**

The carcass characteristics such as ready-to-cook yield, eviscerated weight, and weight of heart, liver, gizzard, giblet, and abdominal fat as a percent of live body weight were not influenced by supplementation of CO and CSB at the levels attempted or by antibiotic supplementation in broilers. The pH, cooking loss, shear force and WHC of meat, appearance, flavor, texture, mouth coating, juiciness and overall acceptability of meat were not influenced by the supplementation of different levels of CO and CSB or by antibiotic supplementation but decreased meat cholesterol level in broilers.

**Conclusion:**

The results indicated that the supplementation of CO and CSB in broiler diet did not alter the carcass characteristics and meat quality parameters except meat cholesterol content in broilers.

## Introduction

There is a need for alternatives to antibiotic growth promoters that ensure animal health and performance without compromising human health. Such alternatives are probiotics, prebiotics, organic acids, phytogenic products, enzymes, betaine, or mixtures of these [1,2]. Some researchers have suggested that organic acids could be used to control intestinal microbial growth [3-5]. Moreover, feeding organic acids are believed to have several beneficial effects such as improving feed conversion ratio, growth performance, enhancing mineral absorption, and speeding recovery from fatigue [6]. Both the organic acids and essential oils separately have been shown to increase production efficiency in broilers, but fewer attempts have been made with a combination of the essential oil and organic acids. The phytogenic additives and organic acids, isolated or associated in broiler diets, improve the nutrient digestibility of the diet, replace the growth-promoting antibiotics, and improve broiler performance [7]. Moreover, the combination of essential oils and acids improves bird’s performance and reduces the cost of production. The synergistic effect of essential oils and acids provides more benefits when combined than individual supplementation [8].

Therefore, investigating the potential synergistic or additive benefits of essential oils with other feed additive combinations including organic acids, probiotics, prebiotics, and enzymes is of far more significance than exploring the individual components.

The objective of this study was to evaluate the carcass characteristics and meat quality parameters and sensory characteristics of meat by supplementation of a combination of cinnamon oil (CO) and sodium butyrate an organic acid as a substitute for the antibiotic (oxytetracycline) of broiler chicken.

## Materials and Methods

### Ethical approval

The present research was conducted after approval of the Institutional Animal Ethics Committee (IAEC/Ab/03/2016), Veterinary College and Research Institute, Namakkal (Tamil Nadu Veterinary and Animal Sciences University [TANUVAS]), Tamil Nadu, India.

### CO and coated sodium butyrate (CSB)

The CSB used in this experiment was encapsulated with a vegetable fatty acid containing 30% SB. The level of coated SB was increased by 3 times since the active compound (SB) in it was 30% (as per the analysis of test sample in this study). CO was purchased as 100% natural oil from M/s. Prime Essentials, Chennai and used in this experiment.

### Experimental design

The biological experiment was conducted with 216-day-old Vencobb-400 broiler chicks. The chicks were wing banded, weighed individually, and assigned randomly to six experimental groups with six replicates per treatment and with six chicks per replicate. Each replicate had equal numbers of male and female chicks. The completely randomized design was followed. The birds were housed in deep litter pens and reared under uniform standard management practices. A 24 h lighting program was provided throughout the experimental period. Chicks were fed with the weighed quantity of isocaloric and isonitrogenous experimental diets and had free access to water. The chicks were vaccinated against Ranikhet disease (RDVB_1_) on the 7^th^ and 21^st^ days and infectious bursal disease on the 14^th^ day of age.

### Experimental diet

Dietary treatments were control (T_1_), ration with 50 ppm of oxytetracycline (T_2_), ration with CO 250 mg/kg and CSB 0.09% (T_3_), ration with CO 250 mg/kg and CSB 0.18% (T_4_), ration with CO 500 mg/kg and CSB 0.09% (T_5_), and ration with CO 500 mg/kg and CSB 0.18% (T_6_). Feed composition and formulation of starter (1-12 days), grower (13-23 days), and finisher (24-35 days) diets were based on Vencobb standard (Table-1).

**Table-1:** Ingredients and nutrient composition of experimental broiler starter, grower, and finisher diets.

Ingredient	Starter	Grower	Finisher
Corn (%)	54.97	56.86	58.84
Soybean meal (%)	38.05	34.40	30.5
Rice bran oil (%)	2.90	4.60	6.30
Calcite (%)	1.35	1.45	1.40
Dicalcium phosphate (%)	1.45	1.35	1.25
Salt (%)	0.40	0.40	0.40
L-Lysine hydrochloride (%)	0.08	0.05	0.03
DL-Methionine (%)	0.20	0.23	0.21
L-Threonine (%)	-	0.01	0.00
Additives and supplements^1-8^*(%)	0.50	0.55	0.77
NSP enzyme^9^(%)	0.0	0.0	0.10
Choline chloride^10^(%)	0.100	0.10	0.10
Soda bicarbonate^11^(g)	0.0	0.0	0.10
Total	100.00	100.00	100.00
Nutrient composition**			
Crude protein (%)	22.50	21.03	20.00
Digestible lysine (%)	1.30	1.20	1.05
Digestible methionine (%)	0.55	0.50	0.45
Metabolizable energy (kcal/kg)^‡^	2950	3124	3150
Calcium (%)	0.94	0.91	0.87
Available phosphorus (%)	0.45	0.42	0.40

* Additives and supplements contained Vitamin A, B_2_, D_3_, and K - for starter: 60 g, for grower: 60 g, and for finisher: 80 g; Vitamin B complex - 20 g; Coccidiostat - 50, 50, and 60 g; Toxin binder - 25, 100, and 100 g; Liver stimulant - 100, 50, and 150 g; Lysoforte - 60 g; Endox dry - 10, 20, and 30 g; and Trace minerals - 150, 150, and 250 g each during starter, grower, and finisher phases. (1) Supplied per kg of diet: Vitamin A - 16,500 IU, Vitamin B2-10 mg, Vitamin D3-3200 IU, and Vitamin K - 2 mg. (2) Supplied per kg of diet: Thiamine - 4 mg, pyridoxine - 8 mg, cyanocobalamine - 40 mcg, Vitamin E - 40 mg, niacin - 60 mg, calcium D pantothenate - 40 mg, folic acid - 4 mg. (3) Coccidiostat containing 25% of 3, 5 Dinitro-ortho-toluamide. (4) Toxin binder containing mixture of silicates, cross-linked insoluble polyvinylpyrrolidone homopolymer, mannan oligosaccharides, yeast cell wall extracts, activated charcoal, XMB factors, multiple organic acids, and lipotropic factor. (5) Liver stimulant containing tricholine citrate, Vitamin B12, inositol, Vitamin E, biotin, selenium, methyl donors, mold inhibitors, and toxin binders. (6) Lysoforte containing lysophospholipids and lysophosphatidylcholine. (7) Endox dry containing ethoxyquin, butylated hydroxyanisole, ethidium diamine tetraacetate, phosphoric acid, citric acid, mono- and di-glycerides, and butylated hydroxyl toluene. (8) Supplied per kg of diet: Manganese - 54 g, zinc - 52 g, iron - 20 g, iodine - 2 g, copper - 2 g, cobalt - 1 g. (9) NSP enzyme each kg containing cellulase - 12,000,000; hemicellulase - 5,400,000; protease - 2,400,000; amylase - 2,400,000; ß-glucanase - 106,000 IU. (10) Choline chloride - 100 g/100 kg. (11) Soda bicarbonate - 10 g/100 kg. Additives (vitamins, trace minerals, toxin binder, and coccidiostat) used in the study were obtained from Virbac Animal Health India, Ltd.

** Calculated value.

‡ ME calculation: 37 × %CP+81 × %EE+35.5 × %NFE

### Carcass characteristics

The effect of supplementation of CO and CSB (T_3_, T_4_, T_5_, and T_6_) on dressing and internal organs as a percentage of live body weight and weight of the abdominal fat in broiler chicken was estimated at the end of the trial (35^th^ day).

### Meat quality parameters

The pH, water-holding capacity (WHC), tyrosine value, shear force value, cooking loss, thiobarbituric acid (TBA) value, sensory evaluation, and muscle cholesterol were estimated at the end of the trial in breast muscle samples.

### Meat pH, WHC tyrosine value, and shear force value

The fresh breast muscle (5 g) collected immediately after slaughter was minced and taken in a beaker containing 45 ml of double glass distilled water (pH - 6.7). The pH of meat was estimated using Oakton multiparameter PCS^™^ 35 tester. The estimation was carried out by adopting the filter paper press method with modification [9]. Accurately weighed (300 mg) muscle sample was kept in between a folded Whatman filter paper. The folded filter paper with meat was then kept in between two glass slides. The muscle tissue was subjected to a downward force by placing a 100 g weight on the top of the upper glass slide for 3 min. The areas of the two resultant wet impressions were expressed in the square centimeter. The tyrosine value was estimated from fresh and stored muscle sample [10]. The tenderness of meat sample was estimated by shear force value. The measurement of shear force value was carried out using a Warner–Bratzler meat shear (G.R. Electric Manufacturing Company, Manhattan, USA). Samples of breast muscle of 5 cm length and 1 cm thickness were taken at 45° angle to obtain parallel alignment of the muscle fibers. The required peak force to attain a shear force value was pre-tested at a speed of 2.0 mm/s, the test speed of 2.0 mm/s and post-test speed of 10.0 mm/second and the load cell was maintained at 25 kg.

### Cooking loss, TBA value, muscle cholesterol, and sensory evaluation of meat

The cooking loss in the meat sample was estimated [11] by placing the muscle piece (50 g) in a polyethylene bag, and its mouth was tied. The bag was immersed in hot water at 75°C for 50 minutes, then placed in running water for 40 min, after which the meat was removed from the bag, mopped dried and weighed. The percentage loss in weight was expressed as cooking loss. The TBA value was estimated using solvent trichloroacetic acid for extraction [12]. The TBA value was expressed as milligram malondialdehyde per kg of chicken meat. The breast**/**thigh muscle samples were chopped and minced with mortar and pestle. The total lipid was extracted from muscle tissue samples [13] using chloroform and methanol (2:1) solutions. The chloroform layer containing cholesterol was separated using separating funnel. The extracted muscle cholesterol was estimated for cholesterol by one-step method [14]. Cholesterol reacts with cholesterol reagent (solution of ferric perchlorate, ethyl acetate, and sulfuric acid) which resulted in the lavender colored complex; the absorbance was measured at 560 nm. Fresh coded breast muscle sample was pressure cooked using a domestic pressure cooker until the internal temperature of the breast muscle was sufficient to cook the meat. The cooked samples were cut into 1 cm slices, and the samples were served to a panel of members (six numbers) selected from the staff members of Veterinary College and Research Institute, Namakkal. They were provided with a scorecard of eight hedonic points to assess the color, appearance, flavor, texture, juiciness, tenderness, and overall acceptability of the meat.

### Statistical analysis

The data were subjected to analysis of variance procedures appropriate for a completely randomized design using the general linear model procedures of SPSS software. Means were compared using Duncan’s multiple range test [15,16].

## Results

### Carcass characteristics

The effect of supplementation of CO and CSB (T_3_, T_4_, T_5_, and T_6_) on dressing and internal organs as a percentage of live body weight and weight of the abdominal fat in broiler chicken is presented in Table-2.

**Table-2:** Effect of supplementation of cinnamon oil and coated sodium butyrate on carcass characteristics (percent), meat quality and muscle cholesterol, and sensory evaluation in broilers.

Experimental treatment								p-value

*Attributes*	T1	T2	T3	T4	T5	T6	SEM	
Carcass characteristics/*Relative organ weight (%)*								
Ready-to-cook weight	75.64	74.09	73.79	73.69	72.69	73.95	0.576	0.589
Eviscerated weight	68.73	67.76	67.53	67.49	66.48	66.35	0.307	0.572
Liver	2.31	2.16	2.24	2.16	2.15	2.27	0.049	0.596
Heart	0.60	0.62	0.57	0.58	0.61	0.66	0.011	0.079
Gizzard	1.90	1.79	1.91	1.82	1.86	2.01	0.029	0.246
Giblets^†^	4.80	4.56	4.72	4.55	4.62	4.95	0.069	0.176
Abdominal Fat	1.37	1.45	1.47	1.52	1.55	1.53	0.050	0.870
Meat quality and muscle cholesterol								
Meat pH	6.05	5.92	6.02	5.95	6.02	6.00	0.032	0.884
WHC (%)	62.05	61.95	62.6	61.48	63.77	61.9	0.316	0.384
Cooking loss (%)	20.58	20.99	21.75	22.88	21.87	21.37	0.235	0.073
Shear force value (kg force/cm^2^)	1.11	1.19	1.31	1.24	1.26	1.28	0.025	0.286
Tyrosine value	20.31	21.40	21.19	21.08	20.88	20.88	0.189	0.696
TBA	0.91	0.95	0.97	0.96	0.92	0.92	0.015	0.854
Muscle cholesterol (mg/dl)	89.71^c^	84.54^b^	79.81^a^	78.35^a^	78.34^a^	79.95^a^	0.817	0.000
Sensory evaluation								
Appearance	6.63	6.50	6.81	6.50	6.56	6.56	0.100	0.958
Flavor	6.25	6.31	6.50	6.38	6.06	6.00	0.096	0.687
Texture	6.56	6.44	6.25	5.94	6.38	6.31	0.096	0.555
Juiciness	6.19	6.13	6.38	6.06	6.50	6.00	0.109	0.783
Mouth coating	6.38	6.31	6.50	6.31	6.44	6.13	0.109	0.955
Overall acceptability	6.31	6.38	6.69	6.50	6.56	6.06	0.076	0.233

T1=Control; T2=T1+antibiotic−OTC at 50 mg/kg; T3=T1+CO - 250 mg/kg+CSB - 0.09%; T4=T1+CO - 250 mg/kg+CSB - 0.18%; T5=T1+CO - 500 mg/kg+CSB - 0.09%; T6=T1+CO - 500 mg/kg+CSB - 0.18%. Each value is a mean of six observations. Mean in a row with different superscripts differ significantly (p<.01). CO=Cinnamon oil, CSB=Coated sodium butyrate, OTC=Oxytetracycline, WHC=Water-holding capacity, TBA=Thiobarbituric acid value.

‡ Giblets consisted of liver without gallbladder, heart without pericardium, and gizzard without inner layer and contents

None of the slaughter parameters were influenced by the low level of CO with either low (T_3_) or high (T_4_) level of CSB and high level of CO with either low (T_5_) or high (T_6_) level of CSB compared to control (T_1_) and antibiotic (T_2_) group in broilers.

### Meat quality parameters

The effect of supplementation of CO and CSB (T3, T4, T5, and T6) on meat pH, WHC, shear force value, cooking loss, tyrosine value, TBA value, and meat cholesterol in broiler chicken is presented in Table-2. The pH and WHC of the breast muscle, cooking loss, shear force value, and TBA value were not influenced by low levels of CO with either low (T_3_) or high (T_4_) level of CSB and high level of CO with either low (T_5_) or high (T_6_) levels of CSB compared to control (T_1_) and antibiotic (T_2_) group in broilers, but the meat cholesterol value is reduced.

### Sensory evaluation

The effect of supplementation of CO and CSB (T_3_, T_4_, T_5_, and T_6_) on the sensory evaluation of meat parameters, namely appearance, flavor, texture, juiciness, mouth coating, and overall acceptability is presented in Table-2. All the parameters of sensory evaluation of meat were found to be comparable between the treatment groups.

## Discussion

In this study, carcass characteristics, meat quality parameters, and sensory evaluation of meat were not influenced by supplementation of a combination of CO and CSB compared to control (T_1_) and antibiotic (T_2_) group in broilers.

### Carcass characteristics

Similar observations pertaining to liver weight [17], abdominal fat percent [18], and dressing percentage [19] were recorded when cinnamon bark oil added in the diet of broilers. However, increased liver weight [18,20] and decreased abdominal fat percent [21] were also recorded in broiler birds supplemented with CO and addition of CO at 100 ppm in broiler diet increased dressing percentage, abdominal fat, liver, heart, and gizzard compared to control. Sodium butyrate did not influence the weight of heart, liver, and abdominal fat. Similar to this finding, some researchers did not observe any changes in heart weight [22]. On the contrary, increased heart weight [23], comparable heart weight by some researchers [24,25], and decreased liver weight were observed due to supplementation of various forms of butyric acid, but gizzard weight was not influenced by the inclusion of butyric acid [22]. Supplementation of thyme oil at 0.2% with citric acid 0.2% in broiler diet did not affect the carcass, liver, gizzard, abdominal fat, total edible parts, and giblets percent [26] whereas an increase in dressing percent, breast, and giblets [27].

The pH and WHC of the breast muscle were not influenced by the combination of CO and CSB. Similarly, supplementation of cinnamon bark powder (CBP) 200 g with antibiotic did not influence meat pH and WHC in broiler, but higher level at 300 g of CBP without antibiotic showed poor WHC, but the same level of CBP with antibiotic had better WHC which was significantly (p<0.05) higher [28]. The increase in the WHC cannot be assigned due to the addition of antibiotic as the same antibiotic addition in control diet resulted in decrease WHC capacity. The addition of CO at 200 ppm in Japanese quail diet did not influence in meat pH [29] and also the microencapsulated sodium butyrate supplementation at 0.04% in broiler diet did not affect meat pH and WHC [30]. Similarly, the addition of SB at different levels did not influence meat pH and WHC in broiler meat [31]. Cooking loss of breast muscle was not influence in the combination of CO and CSB compared to control (T_1_) and antibiotic (T_2_) group in broilers. Similarly, supplementation of CBP at 200 g with an antibiotic in broiler did not influence cooking loss of breast muscle [28]. The CBP addition did not have any difference in cooking loss in quail meat [29]. Cooking loss refers to evaporative or drip losses when meat is cooked. Microencapsulated sodium butyrate supplementation at 0.04% in broiler diet did not influence cooking loss of meat [30]. Shear force value of the breast muscle was not influenced by addition of low level of CO with either low (T_3_) or high (T_4_) level of CSB and high level of CO with either low (T_5_) or high (T_6_) levels of CSB compared to control (T_1_) and antibiotic (T_2_) group in broiler ration. In contrary, supplementation of CBP at 200 and 300 g level decreased the shear force value compared to the control diet [28]. Higher shear force value in the muscle of birds fed with a diet containing antibiotic tylosin both at subtherapeutic and therapeutic condition in broilers [32]. Microencapsulated sodium butyrate supplementation at 0.04% in broiler diet did not affect shear force value [30]. Similar observations were recorded by some of the researchers [31] in addition of sodium butyrate at different levels in broilers. Tyrosine value and TBA of the breast muscle were not influenced by low level of CO with either low (T_3_) or high (T_4_) level of CSB and high level of CO with either low (T_5_) or high (T_6_) level of CSB compared to control (T_1_) and antibiotic (T_2_) groups in broiler ration. A similar observation was recorded by supplementation of thyme and lemongrass oil at different levels in broiler diet [33].

Meat cholesterol value of the breast muscle was significantly (p<0.01) reduced by low levels of CO with either low (T_3_) or high (T_4_) level of CSB and high level of CO with either low (T_5_) or high (T_6_) level of CSB compared to control (T_1_) and antibiotic (T_2_) groups in broiler ration. Similar results were observed by some of the researchers on supplementation of CO at 1000 ppm level in broiler diet [34]. All the parameters of sensory evaluation of meat were found to be comparable between the treatment groups. The CBP at 5% increased the taste and flavor of meat which is in contrary to the present findings [35]. However, similar to the present findings, the supplementation of CBP at 2% did not influence in odor or flavor in broiler meat [36].

## Conclusion

From our results, it is revealed that CO at 250 mg/kg and CSB at 0.09 CBP could be used as alternatives to the antibiotics in broiler feed without compromising their meat quality properties. It is confirmed that combined supplementation of essential oil with acids had potential benefits to the broilers. The quantification of essential oil bioactive components may be necessary to optimize the appropriate dose to use as an alternate antibiotic growth promoter in broiler feeds.

## Authors’ Contributions

MRP and SS were involved in the design of the experiment. GG carried out the broiler trial, data collection, analysis of data, and prepared the first draft of the manuscript. SS and AN assisted GG at all stages of the work. SS, MRP, and RA revised the manuscript. All authors read and approved the final manuscript.
